# Photoinduced Electron Transfer-Promoted Reactions Using Exciplex-Type Organic Photoredox Catalyst Directly Linking Donor and Acceptor Arenes

**DOI:** 10.3390/molecules24244453

**Published:** 2019-12-05

**Authors:** Mugen Yamawaki, Akiko Asano, Toshiki Furutani, Yuki Izumi, Yosuke Tanaka, Kazuyuki Osaka, Toshio Morita, Yasuharu Yoshimi

**Affiliations:** Department of Applied Chemistry and Biotechnology, Graduate School of Engineering, University of Fukui, 3-9-1 Bunkyo, Fukui 910-8507, Japan; infinitybadominnton@yahoo.co.jp (M.Y.); akiko19961995@gmail.com (A.A.); xnx_pxexe_875@yahoo.co.jp (T.F.); izumiyuuki0113@gmail.com (Y.I.); you_sun_31@yahoo.co.jp (Y.T.); sakaoo@u-fukui.ac.jp (K.O.); morita_t@u-fukui.ac.jp (T.M.)

**Keywords:** organic photoredox catalyst, exciplex-type redox catalyst, directly linking between donor and acceptor arenes, photoinduced electron transfer reaction, cross-coupling between alkenes, photoinduced decarboxylation

## Abstract

Directly linked donor and acceptor arenes, such as phenanthrene/naphthalene/biphenyl and 1,3-dicyanobenzene were found to work as photoredox catalysts in the photoreactions of indene, 2,3-dimethyl-2-butene, and 4-methoxyphenylacetic acid. The new stable organic photocatalyst forms an intramolecular exciplex (excited complex) when irradiated in a polar solvent and shows redox catalyst activity, even at low concentrations. To the best of our knowledge, this is the first example of an intramolecular exciplex working as a redox catalyst.

## 1. Introduction

**Scheme 1:**
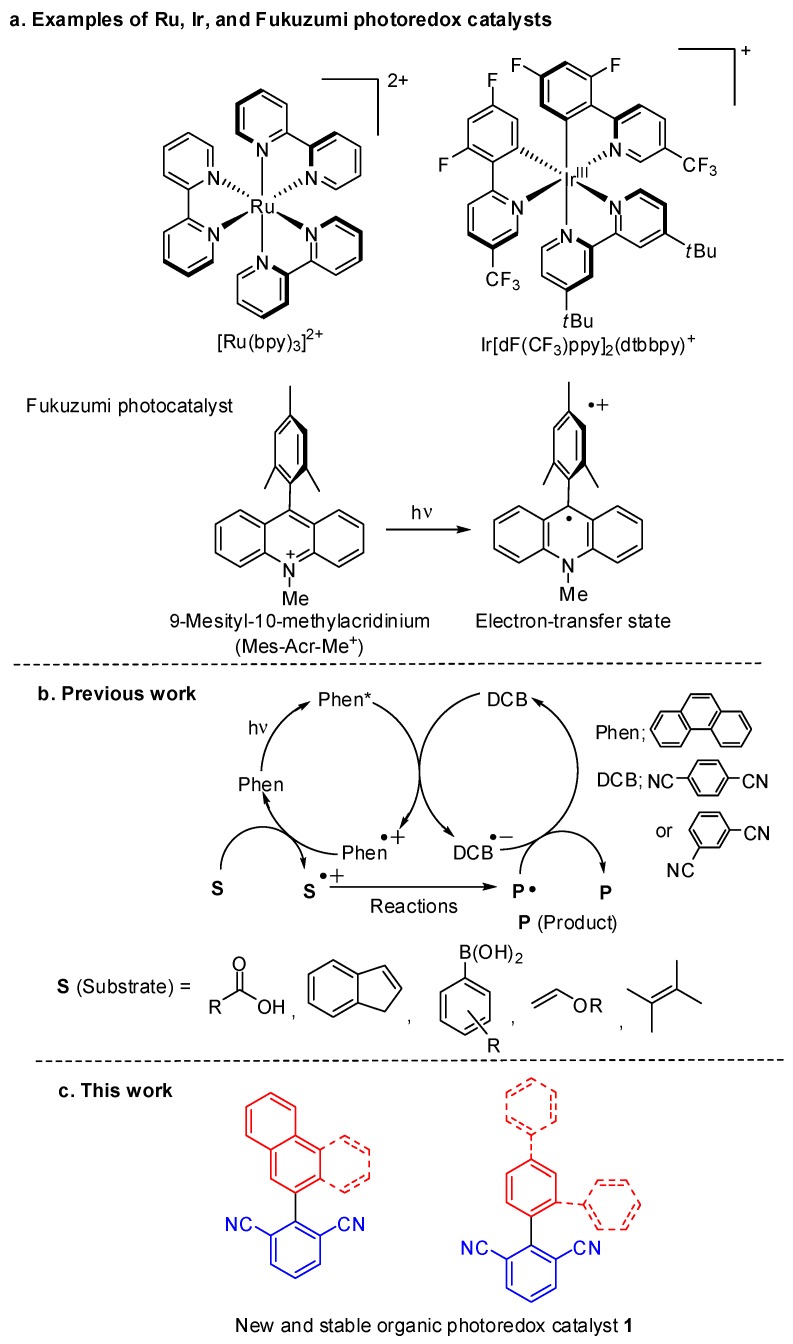
(**a**) Examples of Ru (II), Ir (III), and Fukuzumi photoredox catalysts; (**b**) a summary of our previous work using phenanthrene (Phen) and dicyanobenzene (DCB) as photoredox catalysts; and (**c**) the new type of organic photoredox catalyst discussed in this work.

Photoinduced electron transfer (PET) promoted reactions are a powerful and environmentally friendly tool for the construction of complex organic molecules [1,2,3,4,5] and polymers [6,7] that cannot be prepared by other methods. This is due to the highly reactive radical cation and anion intermediates generated via PET, which facilitate oxidation and reduction reactions under mild conditions (e.g., at room temperature), utilizing light as a clean and traceless reagent. PET-promoted reactions using photoredox catalysts, such as transition-metals (Ru, Ir) [1,2,3,4,5,8] and Fukuzumi photocatalysts [1,2,3,4,5,9], are among the recent trends in organic synthesis (Scheme 1a). The Fukuzumi photocatalyst, in particular—an electron donor–acceptor-linked dyad (9-mesityl-10-methylacridinium ion, Acr^+^-Mes)—acts as an efficient organic photoredox catalyst due to the long-lived electron-transfer state formed upon irradiation. This state can efficiently oxidize and reduce organic molecules to produce the corresponding radical cations and radical anions, respectively. However, these photocatalysts are expensive, have low tolerance for acids and bases, and bear a cationic charge. Thus, a new type of neutral, simple, and stable organic photoredox catalyst is still desirable. We have recently developed PET-promoted reactions of a wide range of functionalized substrates by UV irradiation (313 nm), utilizing the combination of phenanthrene (Phen) as an electron donor and 1,3- or 1,4-dicyanobenzene (DCB) as an electron acceptor (Scheme 1b) [10,11,12,13,14,15,16,17,18]. Herein, we describe the synthesis of neutral and stable organic photoredox catalysts **1**, which directly link with both donor and acceptor arenes, such as Phen/naphthalene (NP)/biphenyl (BP) and 1,3-DCB, and assess their photochemical reactivity (Scheme 1c).

## 2. Results and Discussion

**Scheme 2:**
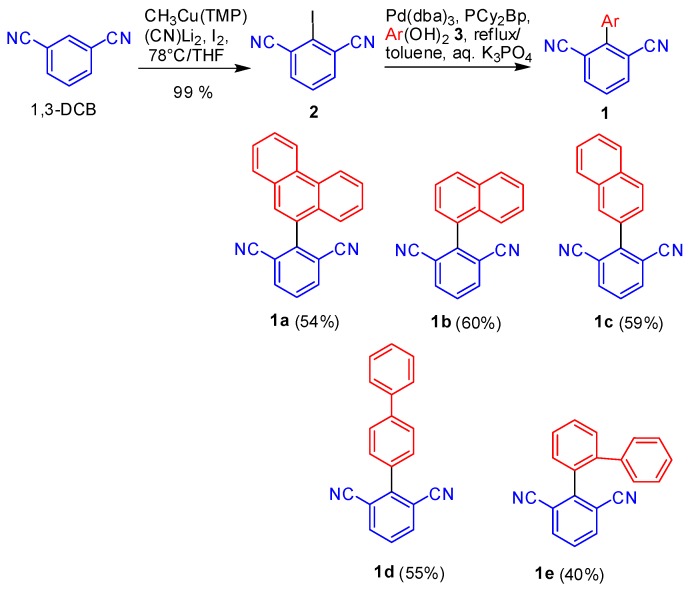
Preparation of photocatalyst **1** from 1,3-DCB and arylboronic acid **3**.

A variety of photocatalysts **1** were prepared from 1,3-DCB and a selection of arylboronic acids **3** (Scheme 2). Initial iodination of 1,3-DCB by CH_3_Cu(TMP)(CN)Li_2_ and I_2_ yielded 1,3-dicyano-2-iodobenzene **2** in a 99% yield [19]. Suzuki–Miyaura coupling [20] of 9-phenanthrylboronic acid **3a** with **2** in the presence of Pd(dba)_3_ and (4-biphenyl)dicyclohexylphosphine (PCy_2_Bp) produced photocatalyst **1a** in moderate yield (52%). Similar coupling reactions of 1-naphthylboronic acid **3b**, 2-naphthylboronic acid **3c**, 4-biphenylboronic acid **3d**, and 2-biphenylboronic acid **3e** provided photocatalysts **1b**–**e** in moderate yields.

**Figure 1:**
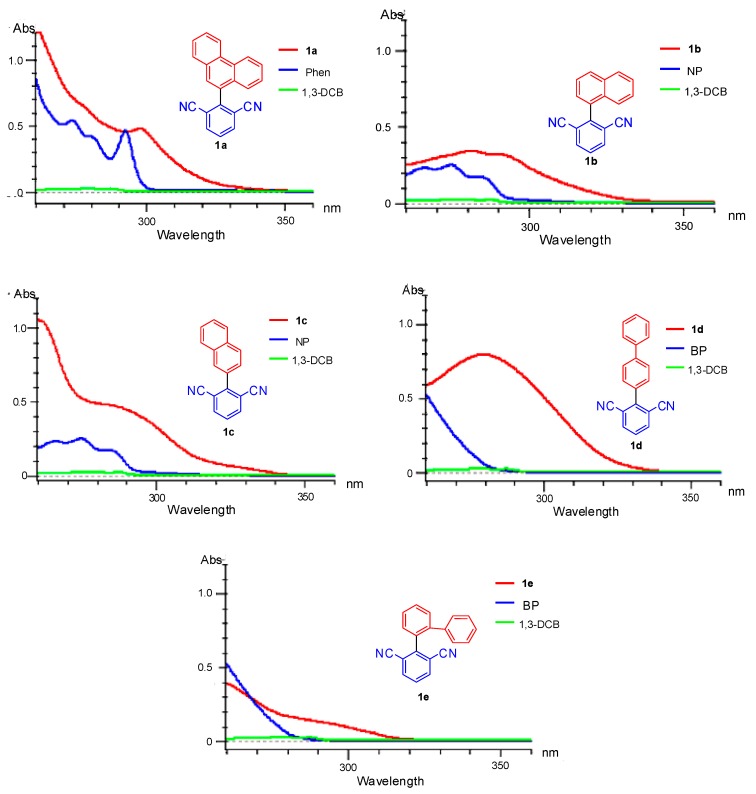
Absorption spectra of **1a**–**e**, Phen, NP, and BP (5 × 10^−5^ M) in an aqueous acetonitrile solution (CH_3_CN/H_2_O = 9:1, *v*/*v*).

**Figure 2:**
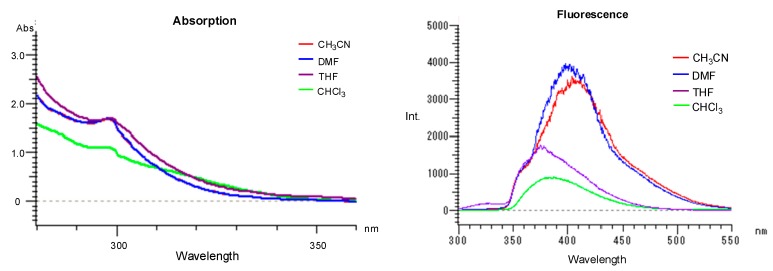
Absorption and fluorescence spectra of **1a** in CH_3_CN, DMF, THF, and CHCl_3_ solution (5 × 10^−5^ M) excited at 260 nm.

**Figure 3:**
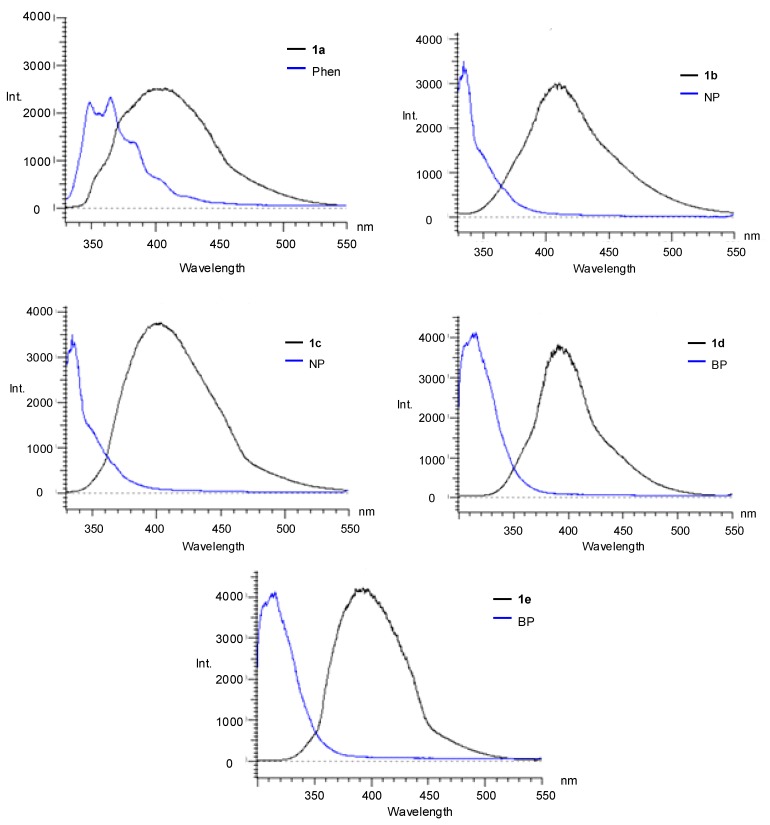
Fluorescence of **1a**–**e**, Phen, NP, and BP (5 × 10^−5^ M) excited at 260 nm in an aqueous acetonitrile solution (CH_3_CN/H_2_O = 9:1, *v*/*v*).

**Figure 4:**
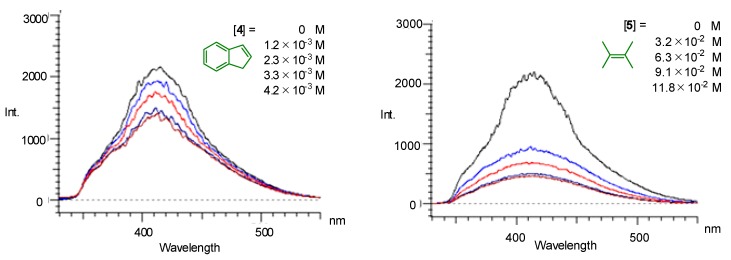

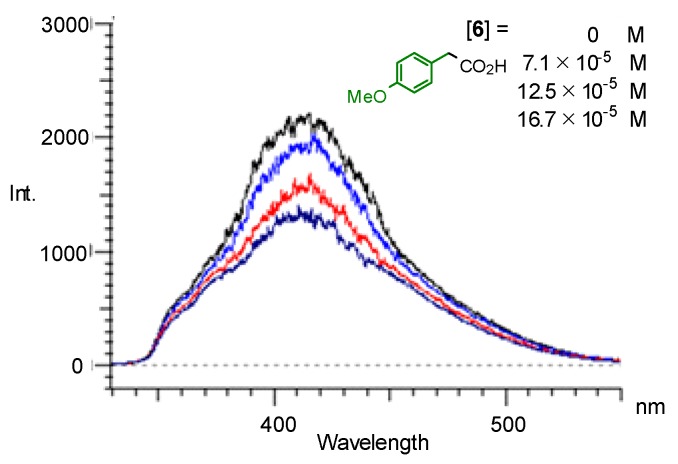
Fluorescence quenching of **1a** (5 × 10^−5^ M) by **4**, **5**, and **6** excited at 260 nm in an aqueous acetonitrile solution (CH_3_CN/H_2_O = 9:1, *v*/*v*).

Next, the UV absorption spectra of photocatalysts **1a**–**e** were measured in aqueous acetonitrile solution (CH_3_CN/H_2_O = 9:1, *v*/*v*) and are shown in Figure 1. The absorption spectrum of **1a** (red) overlapped with that of Phen (blue) and 1,3-DCB (green), and showed an extended absorption range compared to that of Phen, indicating the existence of a π–π interaction between the Phen and DCB moieties in the ground state of **1a** [21,22,23]. A similar trend of extended absorption ranges was observed for other catalysts **1b**–**e**, bearing naphthyl and biphenyl units. These results indicated that the rotation of the aryl-aryl σ-bonds in **1** is not completely restricted by the cyano substituents and that π-π interactions exist between the donor and acceptor arene units within **1**. In fact, the UV absorption spectrum of the less sterically hindered **1d** showed stronger absorption at long wavelengths, as compared to that of the bulkier **1a** and **1e**. This could be attributed to the stronger intramolecular interaction between the aromatic rings, due to the smooth rotation of the aryl–aryl σ-bonds [9,21,22,23]. Solvent polarity did not have any notable effect on the absorption spectra of **1a** (Figure 2). In contrast, the use of more polar solvents, such as CH_3_CN and DMF, caused broadening of the observed fluorescence spectra, which is characteristic of an intramolecular exciplex (Figure 2). Compared to the fluorescence of Phen, the quenching fluorescence of the phenanthryl part by the DCB part in **1a** and the intramolecular exciplex fluorescence were obtained in an aqueous acetonitrile solution (Figure 3). A similar quenching and appearance of fluorescence in **1b**–**e** was observed, indicating that intramolecular exciplexes were formed (Figure 3). In addition, the exciplex fluorescence of **1a** may be quenched by indene **4**, 2,3-dimethyl-2-butene **5**, and (4-methoxyphenyl)acetic acid **6**, for which the Stern-Volmer constants kqτ were estimated to be 1.2 × 10^−1^, 2.1 × 10^−2^, and 8.5 × 10^−1^/M, respectively (Figure 4). These results indicated that the donor and acceptor arene parts in **1** interacted, both in the ground and excited states, through the rotation of the aryl–aryl σ-bond and formed intramolecular exciplexes in a polar solvent, which were quenched by **4**, **5**, and **6**.

**Scheme 3:**
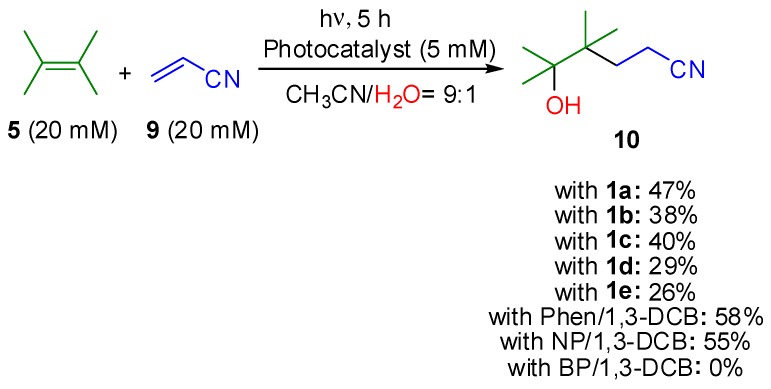
Photoinduced cross-coupling reaction of **5** and **9** by photoredox catalysts.

**Scheme 4:**
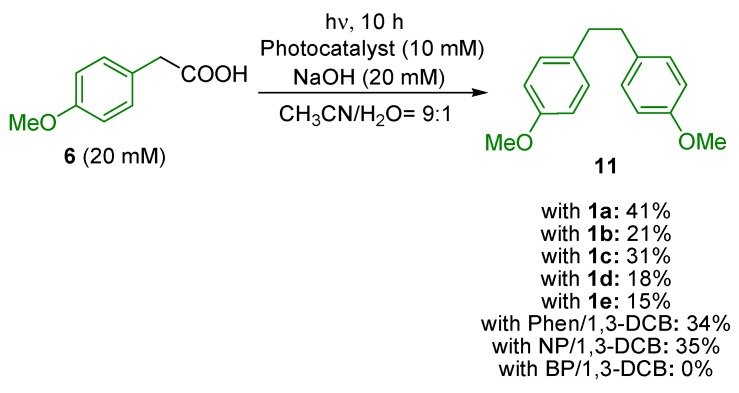
Photoinduced decarboxylative radical dimerization of **6** by photoredox catalysts.

We attempted to evaluate the photochemical reactivity of photocatalyst **1** using indene **4** [16] (Table 1), 2,3-dimethyl-2-butene **5** [18] (Scheme 3), and (4-methoxyphenyl)acetic acid **6** [10,11,12,13,14,15] (Scheme 4) as substrates. Reactions were carried out in an aqueous acetonitrile solution (CH_3_CN/H_2_O = 9:1, *v*/*v*), under an argon atmosphere, under irradiation by a 100 W high-pressure mercury lamp in Pyrex vessels (18 × 180 mm) at room temperature. The irradiation of **4** (20 mM) for 10 h, in the presence of **1a** as a photoredox catalyst, exclusively afforded alcohol **7** in a 49% yield through the formation of a radical cation of **4** via PET between the excited state of **1a** and **4** [16] (Entry 1, Table 1), along with the unidentified products. The quantum yield of this photoinduced reaction of **4** using **1a** is about 0.01. In the absence of **1a**, the photoreaction of **4** under the same conditions yielded only the [2 + 2] cycloadduct **8** through dimerization between a molecule of **4** in the excited state with another in the ground state (Entry 2). Using a combination of Phen and 1,3-DCB as photocatalysts, the alcohol **7** was yielded (50%), as observed in Entry 1, along with a small amount of **8** (6%) (Entry 3), indicating that the efficiency of **1a** in the photoreaction of **4** is comparable to that of the Phen/1,3-DCB system. This suggests that the appearance of the longer absorption of **3a** prevented dimerization of **4**. The use of only Phen or 1,3-DCB as the photocatalyst in the photoreaction did not work well (Entries 4 and 5). Decreasing the concentration of **1a** led to a decrease in the yield of **7** and an increase in the yield of **8** (Entries 6–9) because of the gradually diminished absorption of **1a**. It should be noted, however, that **7** was obtained even when using a very low concentration of **1a** (0.1 mM, Entry 9). When the intermolecular Phen/1,3-DCB system was subjected to the same low concentration conditions (0.1 mM) only dimer **8** was observed (Entry 10). Thus, even at low concentrations, **1a** works as a more efficient photoredox catalyst than the Phen/1,3-DCB system because of the intramolecular system. The use of **1b**,**c** bearing a naphthyl group also yielded alcohol **7** in similar yields to those observed for **1a** and the NP/1,3-DCB system (Entries 11–13). Interestingly, similar photoreactions of **1d** and **1e**, bearing biphenyl groups, afforded **7** along with **8** (Entries 15 and 16), whereas the intermolecular BP/1,3-DCB system exclusively gave **8** because it does not absorb at 313 nm (Entry 14). Direct linking of BP and 1,3-DCB through σ-bonding led to longer absorption, which lead to them working as a photoredox catalyst by 313 nm light-irradiation.

The photoreaction of 2,3-dimethyl-2-butene **5** (20 mM) with acrylonitrile **9** (20 mM) in the presence of **1a** (5 mM) for 5 h in aqueous acetonitrile solution (CH_3_CN/H_2_O = 9:1, *v*/*v*) provided adduct **10** in moderate yield (47%) through the formation of a radical cation of **5** [18] (Scheme 3), along with oligomeric products. Catalysts **1b**–**e** also worked for the photoreaction of **5** with **9** to furnish **10**, although the BP/1,3-DCB system did not act as a photoredox catalyst under these photochemical conditions.

Recently, photoinduced decarboxylation of carboxylic acids using the Phen/DCB system has been established as a powerful method for the generation of alkyl radicals that can react with a variety of radical acceptors to provide the respective products in high yields [10,11,12,13,14,15]. However, photoinduced decarboxylation of carboxylic acids, such as *N*-Boc valine (Boc = *t*-butoxycarbonyl) using **1**, were unsuccessful and the starting carboxylic acids were almost recovered. The use of (4-methoxyphenyl)acetic acid **6** (20 mM) as the substrate, which had a more easily oxidizable aromatic ring, with **1a** (10 mM) in the presence of NaOH (20 mM) yielded dimer **11**. This reaction may proceed through photoinduced decarboxylation, following the formation of a radical cation localized in the aromatic part of **6**, via PET with **1a**. Thus, PET-promoted decarboxylation using **1** required substrates bearing oxidizable aromatic rings.

**Scheme 5:**
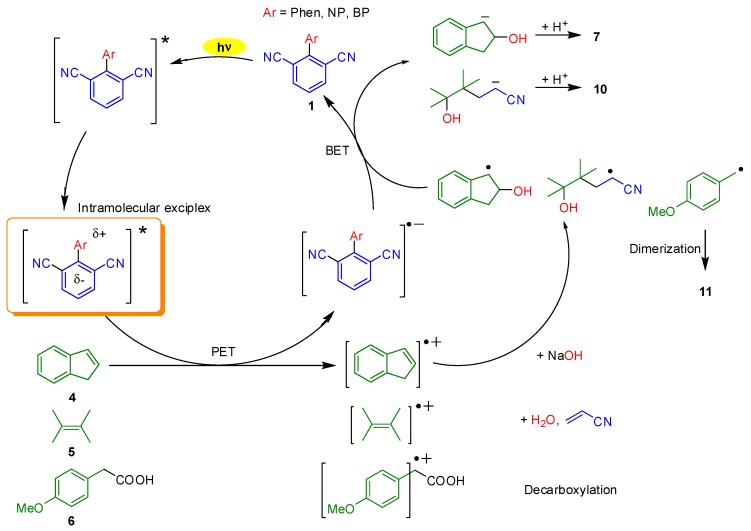
The proposed mechanism of **4**–**6** using **1**.

Based on these results, a plausible mechanism is proposed, as shown in Scheme 5. First, the excited state of **1** formed by absorption at 313 nm leads to the formation of an intramolecular exciplex in a polar solvent. Even though substrates, such as **4**–**6** have higher oxidation potentials than those of the corresponding radical cations of the arene moieties in **1a**–**e** (**4**: +1.66 V vs. SCE in acetonitrile [24], **5**: +2.08 V [18], **6**: methoxybenzene +1.81 V [24], Phen: +1.50 V [13], NP: +1.70 V [13], BP: +1.85 V [18]), PET occurs between the exciplex and **4**–**6** proceeds to form both the corresponding radical cations of **4**–**6** and the radical anion of **1**. This may occur due to the formation of a π-complex between the exciplex and substrates **4**–**6**, which is considered to promote electron transfer [18]. These radical cations react with H_2_O and an alkene or induce decarboxylation to generate the corresponding radicals. Back electron transfer (BET) from the radical anions of **1** to the thusly formed substrate radicals generate anions, followed by protonation, to yield the electronically neutral products **7** and **10**. In the case of **6**, the radical generated via decarboxylation dimerizes to give **11** and BET from the generated radical anion of **1** does not occur, indicating that **1** does not work as a catalyst in this photodecarboxylation. In general, the intramolecular exciplex is considered an important intermediate to cycloaddition [25,26]. However, in this case, the intramolecular exciplex of **1** can work as a redox catalyst for substrates containing alkene or aromatic ring functional groups.

## 3. Materials and Methods

General Information: All reagents and solvents were used as received from commercial suppliers. IR spectra were recorded on an FT-IR spectrometer. ^1^H NMR spectra were recorded in CDCl_3_ containing tetramethylsilane as an internal standard and were acquired on a 500 MHz spectrometer. ^13^C{^1^H} NMR spectra were acquired on a 125 MHz spectrometer. High-resolution mass spectra were obtained using a double-focusing magnetic sector mass spectrometer coupled with FAB. The UV-light source was a Riko UV-100HA high-pressure (100 W) mercury arc. Pyrex vessels (18 mm × 180 mm) were directly attached to the light source (λ > 280 nm, Photocatalyst **1** mainly absorbs at 313 nm light). Column chromatography was performed on Wakogel C-300, particle size 45–75 µm.

**General procedure for synthesis of photocatalyst 1**

To a solution of 2,2,6,6-tetramethylpiperidine (0.68 mL, 4.0 mmol) in 3 mL of anhydrous THF, *n*-BuLi (2.44 M *n*-hexane solution, 1.64 mL, 4.0 mmol) was added at −78 °C under an Ar atmosphere. The mixture was stirred for 30 min at 0 °C. The resulting solution was added to a suspension of copper cyanide (183 mg, 2.0 mmol) in 5 mL of anhydrous THF at −78 °C under an Ar atmosphere. The mixture was stirred at 0 °C for 30 min to give a solution of (TMP)_2_CuCNLi_2_ (2.0 mmol). 1,3-Dicyanobenzene (0.105 g, 1.0 mmol) in anhydrous THF (5 mL) was then added to the resulting mixture at −78 °C under an Ar atmosphere, and the mixture was stirred for 3 h at 0 °C. I_2_ (1.79 g 7.0 mmol) in anhydrous THF (5 mL) was slowly added to the mixture at −78 °C, and the resulting mixture was stirred at room temperature for 16 h before quenching with saturated aq. NH_4_Cl and NaHS_2_O_3_. The resultant reaction mixture was extracted with AcOEt and dried over Na_2_SO_4_, and volatiles were removed by evaporation. The crude product was purified by silica-gel column chromatography using hexane/EtOAc = 9:1 as the eluent to yield 1,3-dicyano-2-iodobenzene **2** as a white solid. Compound **2** has been previously reported [19]. ^1^H and ^13^C{^1^H} NMR spectra for all compounds **1**, **2**, **7**, **8**, **9**, **10**, and **11** can be found in the Supplementary Materials.

***1,3-Dicyano-2-iodobenzene 2*:** White solid; ^1^H NMR (500 MHz, CDCl_3_) δ 7.80 (d, *J* = 7.4 Hz, 2H), 7.65–7.62 (m, 1H); ^13^C{^1^H} NMR (125 MHz, CDCl_3_) δ 137.2, 129.1, 123.4, 118.2, 103.7.

A solution of **2** (0.10 g, 0.39 mmol), arylboronic acid **3** (0.47 mmol), K_3_PO_4_ (0.020 g, 0.094 mmol), Pd_2_(dba)_3_ (0.014 mg, 1.7 mol %), and (4-biphenyl)dicyclohexylphosphine (PCy_2_Bp) (0.014 mg, 3.4 mol%) in toluene (6 mL) was refluxed at 130 °C for 24 h. After the starting material **2** was consumed, the solvent was removed by evaporation in vacuo. The crude product was purified by silica-gel column chromatography using hexane/EtOAc = 9:1 as the eluent to yield photocatalyst **1** as a white solid.

***1,3-Dicyano-2-(9-phenanthryl)benzene 1a***: White solid, m.p. 196–197 °C; IR (KBr, cm^−1^) 3065, 2227; ^1^H NMR (500 MHz, CDCl_3_) δ 8.82 (d, *J* = 8.0 Hz, 1H), 8.77 (d, *J* = 8.6 Hz, 1H), 8.06 (d, *J* = 8.0 Hz, 2H), 7.95 (d, *J* = 8.0 Hz, 1H), 7.78–7.66 (m, 5H), 7.57 (t, *J* = 14.5 Hz, 1H), 7.29 (d, *J* = 8.0 Hz, 1H); ^13^C{^1^H} NMR (125 MHz, CDCl_3_) δ 148.0, 136.7, 131.2, 130.8, 130.7, 129.6, 129.4, 129.3, 129.0, 128.1, 127.4, 127.3, 125.1, 123.5, 122.8, 116.2, 116.1; HRMS (FAB) calcd. for (M + H)^+^ C_22_H_12_N_2_: 305.1079, found: 305.1070.

***1,3-Dicyano-2-(1-naphthyl)benzene 1b***: White solid, m.p. 195–196 °C; IR (KBr, cm^−1^) 3075, 2238; ^1^H NMR (500 MHz, CDCl_3_) δ 8.05 (d, *J* = 8.0 Hz, 2H), 7.98 (d, *J* = 8.0 Hz, 1H), 7.79 (d, *J* = 7.4 Hz, 1H), 7.73–7.70 (m, 1H), 7.65–7.60 (m, 1H), 7.58–7.55 (m, 1H), 7.50–7.48 (m, 2H), 7.28 (d, *J* = 8.6 Hz, 1H); ^13^C{^1^H} NMR (125 MHz, CDCl_3_) δ 148.1, 137.3, 136.7, 131.9, 131.0, 130.9, 129.2, 129.0, 127.8, 127.4, 126.7, 125.3, 124.2, 116.2, 116.1; HRMS (FAB) calcd. for (M + H)^+^ C_22_H_12_N_2_: 255.0923, found: 255.0902.

***1,3-Dicyano-2-(2-naphthyl)benzene 1c***: White solid, m.p. 176–177 °C; IR (KBr, cm^−1^) 3075, 2236; ^1^H NMR (500 MHz, CDCl_3_) δ 8.05–8.01 (m, 4H), 7.96–7.93 (m, 2H), 7.65–7.57 (m, 4H); ^13^C{^1^H} NMR (125 MHz, CDCl_3_) δ 148.9, 137.1, 129.4, 129.0, 128.6, 128.4, 127.9, 127.6, 127.0, 125.8, 116.7, 114.7; HRMS (FAB) calcd. for (M + H)^+^ C_22_H_12_N_2_: 255.0923, found: 255.0903.

***1,3-Dicyano-2-(4-biphenyl)benzene 1d***: White solid, m.p. 180–181 °C; IR (KBr, cm^−1^) 3060, 2227; ^1^H NMR (500 MHz, CDCl_3_) δ 8.01 (d, *J* = 8.0 Hz, 2H), 7.78 (d, *J* = 8.0 Hz, 2H), 7.67 (d, *J* = 7.4 Hz, 2H), 7.63–7.60 (m, 3H), 7.50–7.47 (m, 2H), 7.42–7.39 (m, 1H); ^13^C{^1^H} NMR (125 MHz, CDCl_3_) δ 148.6, 143.1, 140.0, 137.2, 132.9, 129.7, 128.9, 128.4, 127.9, 127.7, 127.3, 116.7, 114.4; HRMS (FAB) calcd. for (M + H)^+^ C_20_H_13_N_2_: 281.1079, found: 281.1068.

***1,3-Dicyano-2-(2-biphenyl)benzene 1e***: White solid, m.p. 187–188 °C; IR (KBr, cm^−1^) 3065, 2238; ^1^H NMR (500 MHz, CDCl_3_) δ 7.80–7.77 (m, 2H), 7.63–7.60 (m, 1H), 7.55–7.52 (m, 2H), 7.47–7.44 (m, 1H), 7.40–7.39 (m, 1H), 7.22–7.16 (m, 5H); ^13^C{^1^H} NMR (125 MHz, CDCl_3_) δ 149.5, 141.9, 139.5, 137.2, 136.3, 133.1, 130.8, 130.5, 129.8, 129.7, 129.1, 128.2, 128.0, 127.8, 127.3, 123.4, 118.2, 116.3, 115.6; HRMS (FAB) calcd. for (M + H)^+^ C_20_H_13_N_2_: 280.1079, found: 280.1068.

**General procedure for the photoreaction of 4, 5, and 6 with 1**

An aqueous CH_3_CN solution (CH_3_CN 45 mL, H_2_O 5 mL) of **1** (10 mM), **4** (20 mM), NaOH (20 mM) in Pyrex vessels (18 mm x 180 mm) was purged with Ar for 10 min. The mixture was irradiated with a 100 W high-pressure mercury lamp for 10 h and then the solvent was removed under reduced pressure. The crude product was purified by silica-gel column chromatography using hexane/EtOAc = 9:1 as the eluent to yield alcohol **7** along with dimer **8**. Similar photoreactions of **5** and **6** were carried out under the similar conditions to yield **10** and **11**.

***2-Hydroxyindane 7***: Compound **9** has been previously reported [16]. White solid, ^1^H NMR (500 MHz, CDCl_3_) δ 7.21 (m, 4H), 4.70 (s, 1H), 3.22 (dd, *J* = 8.3, 3.0 Hz, 2H), 2.92 (dd, *J* = 8.3, 1.2 Hz, 2H), 1.68 (d, *J* = 5.2 Hz, 1H); ^13^C{^1^H} NMR (125 MHz, CDCl_3_) δ 140.8, 126.7, 125.0, 73.2, 42.7

***Cyclobutadiindene 8***: Compound **8** has been previously reported [16]. White solid, ^1^H NMR (500 MHz, CDCl_3_) δ 7.41 (d, *J* = 7.4 Hz, 2H), 7.31 (d, *J* = 6.9 Hz, 2H), 7.29–7.22 (m, 4H), 3.70 (d, *J* = 5.2 Hz, 2H), 3.19 (dd, *J* = 8.5, 3.8 Hz, 1H), 2.94 (d, *J* = 8.3 Hz, 2H), 2.78–2.76 (m, 2H); ^13^C{^1^H} NMR (125 MHz, CDCl_3_) δ 146.6, 144.0, 126.9, 126.8, 125.5, 125.2, 54.0, 43.2, 39.4

***5-Hydroxy-4,4,5-trimethylhexanenitrile 10***: Compound **10** has been previously reported [18]. Colorless oil, ^1^H NMR (500 MHz, CDCl_3_): δ 2.41 (t, *J* = 8.0 Hz, 2H), 1.80 (t, *J* = 8.0 Hz, 2H), 1.39 (s(br), 1H), 1.20 (s, 6H), 0.92 (s, 6H); ^13^C{^1^H} NMR (125 MHz, CDCl_3_): δ 121.2, 75.5, 39.9, 33.8, 25.9, 21.9, 13.6.

***1,2-Di(4-methoxyphenyl)ethane 11***: Compound **11** has been previously reported [27]. White solid, ^1^H NMR (500 MHz, CDCl_3_) δ 7.08 (d, *J* = 8.2 Hz, 4H), 6.82 (d, *J* = 8.2 Hz, 4H), 3.80 (s, 6H), 2.83 (s, 4H); ^13^C{^1^H} NMR (125 MHz, CDCl_3_): δ 157.9, 134.1, 129.4, 113.8, 55.3, 37.4.

## 4. Conclusions

This study has led to the development of PET-promoted reactions using a new class of organic photoredox catalysts **1** wherein donor and acceptor arenes are directly linked. The intramolecular exciplex of **1** functions as a photoredox catalyst for the photoreactions of **4**, **5**, and **6** in an aqueous acetonitrile solution, even at low concentrations. Further investigations on the application of catalyst **1** to other PET-promoted reactions and the elucidation of the detailed mechanism are underway.

## Figures and Tables

**Table 1:**
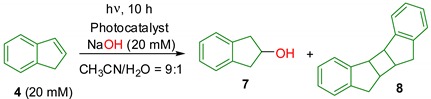
Photoreaction of indene **4** with a photoredox catalyst.

Entry	Photocatalyst	Yield of 7/% ^1^	Yield of 8/% ^1^
1	**1a** (10 mM)	49	0
2	none	0	60
3	Phen (10 mM), 1,3-DCB (10 mM)	50	6
4	Phen (10 mM)	0	trace
5	1,3-DCB (10 mM)	trace	19
6	**1a** (5 mM)	42	3
7	**1a** (1 mM)	34	14
8	**1a** (0.5 mM)	30	25
9	**1a** (0.1 mM)	10	37
10	Phen (0.1 mM), 1,3-DCB (0.1 mM)	0	32
11	NP (10 mM), 1,3-DCB (10 mM)	45	trace
12	**1b** (10 mM)	45	trace
13	**1c** (10 mM)	41	0
14	BP (10 mM), 1,3-DCB (10 mM)	0	24
15	**1d** (10 mM)	36	14
16	**1e** (10 mM)	27	17

^1^ Isolated yield.

## Supplementary Materials

^1^H and ^13^C{^1^H} NMR spectra for all compounds **1**, **2**, **7**, **8**, **9**, **10**, and **11**.
