# Supplementary material for: Photoinduced Electron Transfer-Promoted Reactions Using Exciplex-Type Organic Photoredox Catalyst Directly Linking Donor and Acceptor Arenes
Source: Molecules. 2019 Dec 5;24(24):4453. doi: 10.3390/molecules24244453 (PMC6943656; doi:10.3390/molecules24244453)
Supplement: Supplementary file 1 [file molecules-24-04453-s001.pdf]

Supplementary Materials

Photoinduced Electron Transfer-Promoted Reactions Using Exciplex-Type Organic Photoredox Catalyst Directly Linking Donor and Acceptor Arenes

Mugen Yamawaki, Akiko Asano, Toshiki Furutani, Yuki Izumi, Yosuke Tanaka, Kazuyuki Osaka, Toshio Morita, Yasuharu Yoshimi*

Department of Applied Chemistry and Biotechnology, Graduate School of Engineering, University of Fukui, 3-9-1 Bunkyo, Fukui 910-8507, Japan

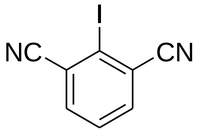

2

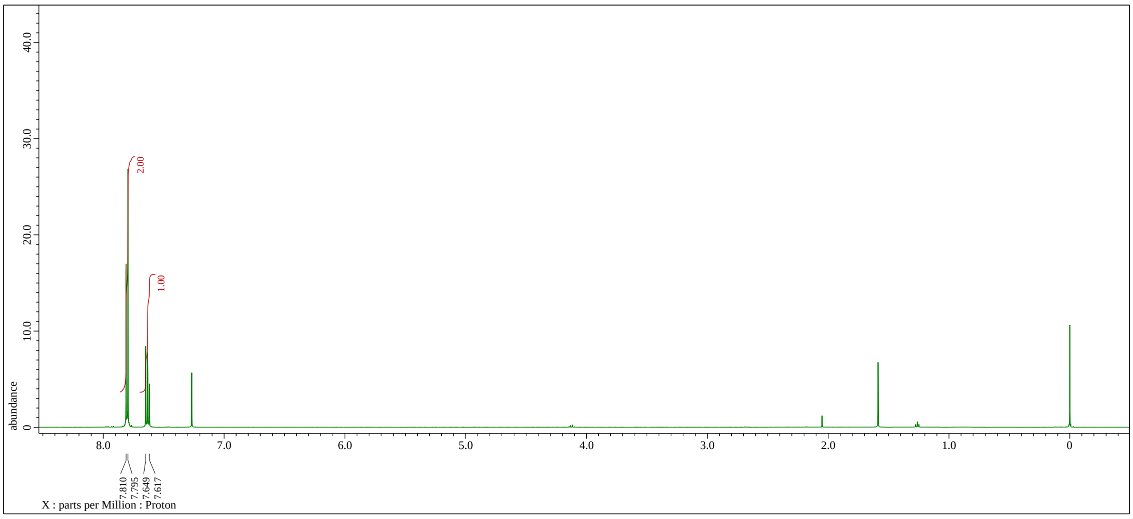

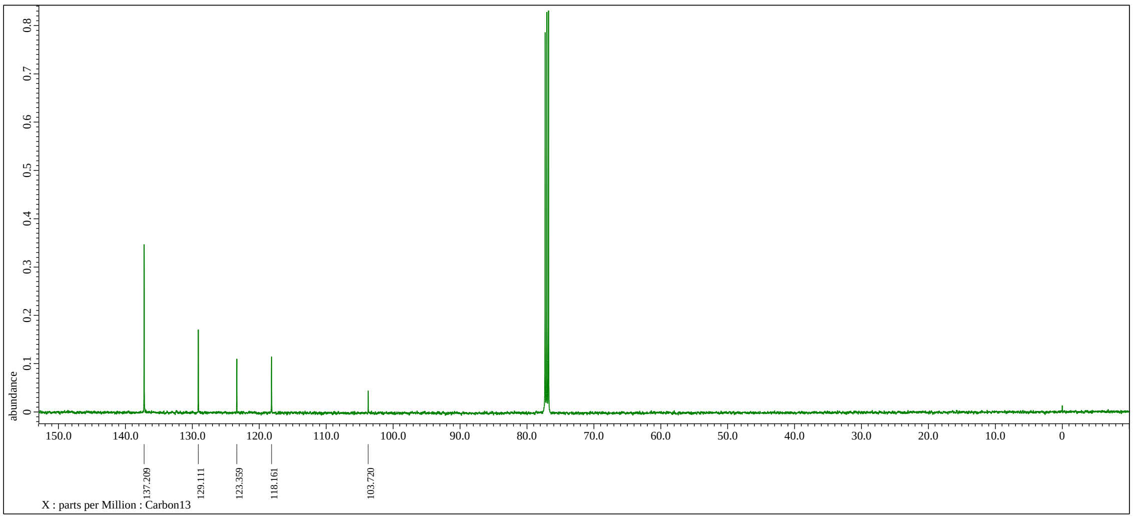

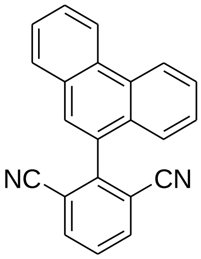
NC
CN

1a

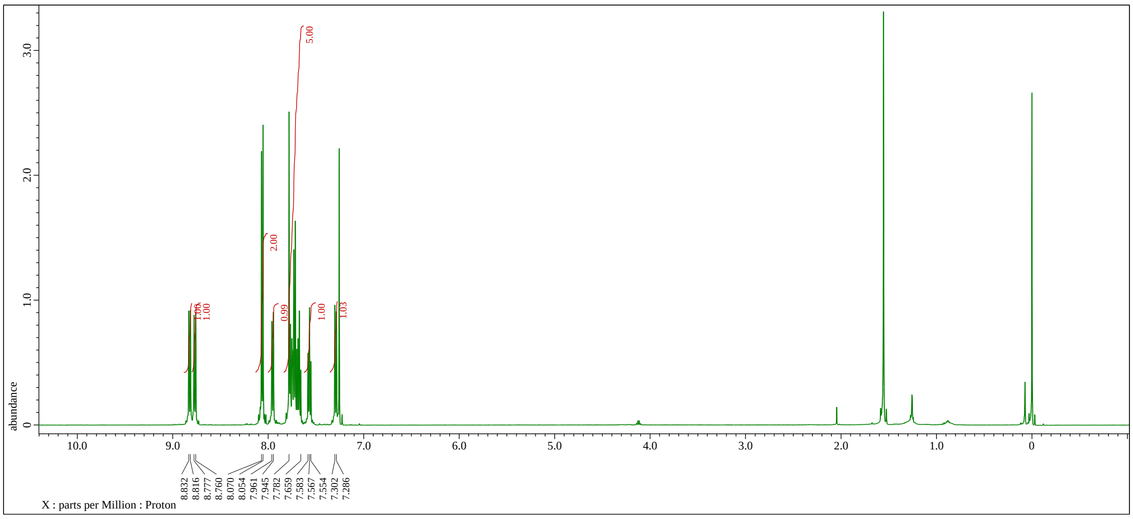
abundance
X : parts per Million : Proton
1.00
1.00
2.00
0.99
5.00
1.00
1.03
8.832
8.816
8.777
8.760
8.070
8.054
7.961
7.945
7.782
7.659
7.583
7.567
7.554
7.302
7.286

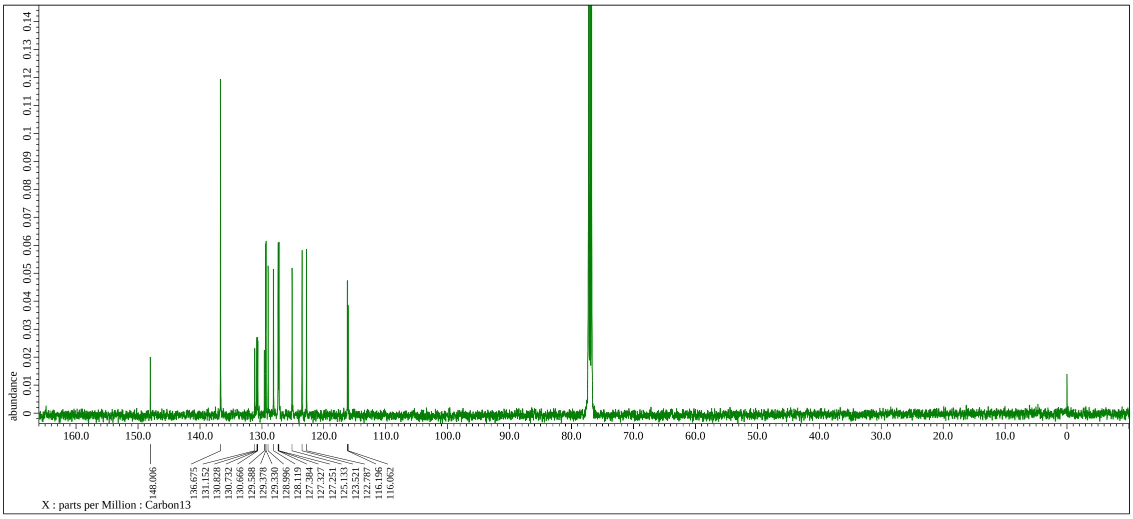
abundance
X : parts per Million : Carbon13
148.006
136.675
131.152
130.828
130.732
130.666
129.588
129.378
129.330
128.996
128.119
127.384
127.327
127.251
125.133
123.521
122.787
116.196
116.062

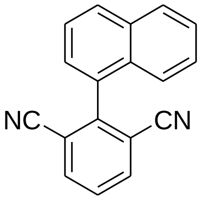

1b

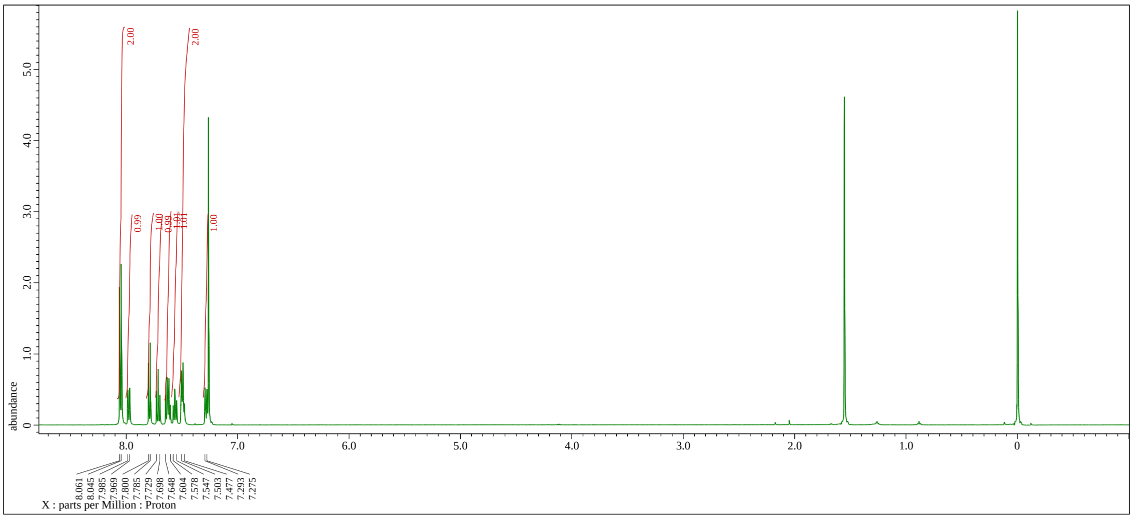

abundance
X : parts per Million : Proton

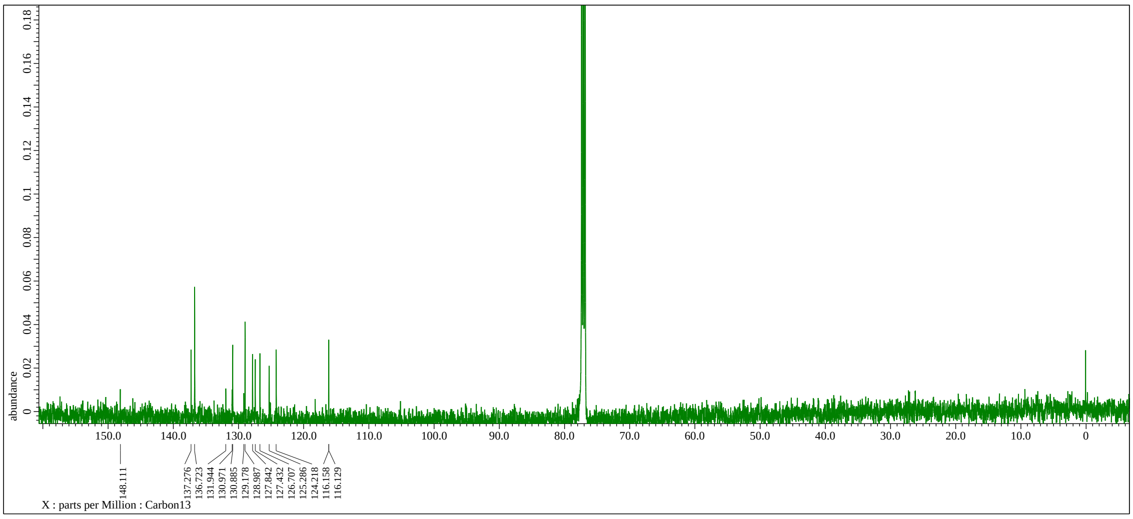

abundance
X : parts per Million : Carbon13

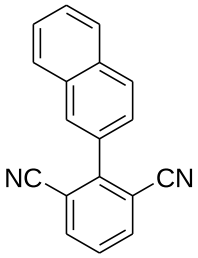
NC
CN

1c

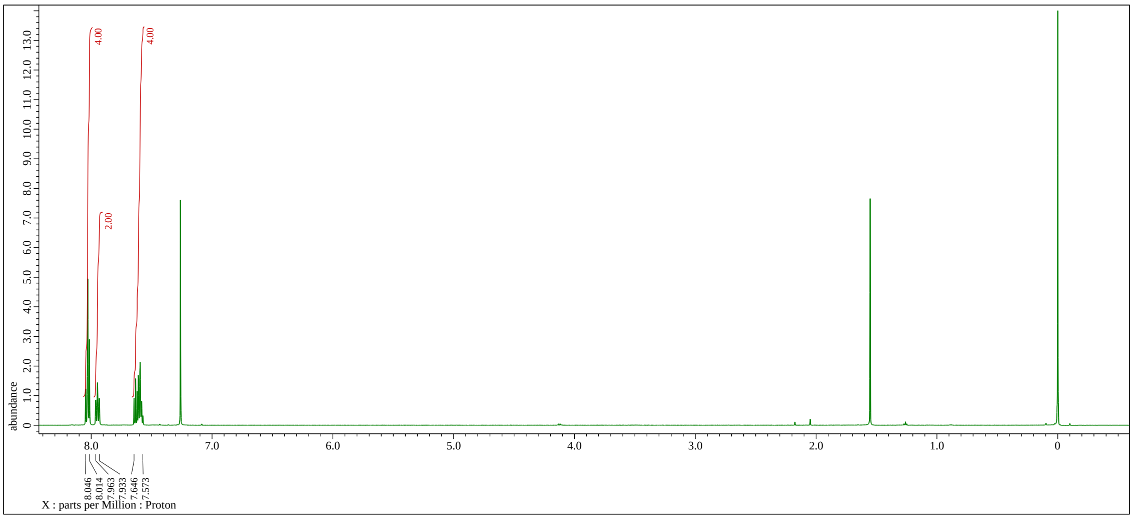
abundance
4.00
4.00
2.00
8.046
8.014
7.963
7.933
7.646
7.573
X : parts per Million : Proton

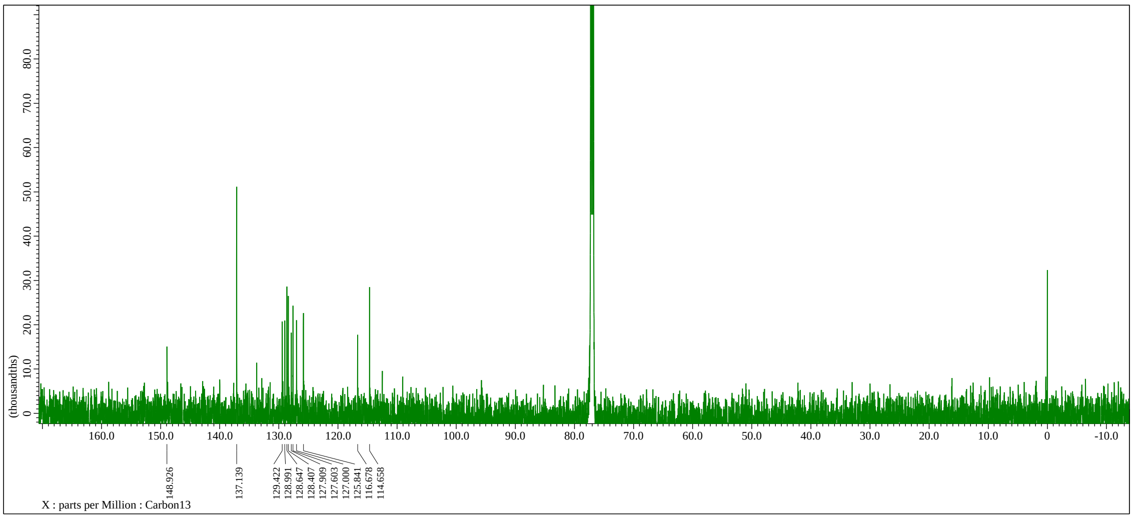
(thousandths)
148.926
137.139
129.422
128.991
128.647
128.407
127.909
127.603
127.000
125.841
116.678
114.658
X : parts per Million : Carbon13

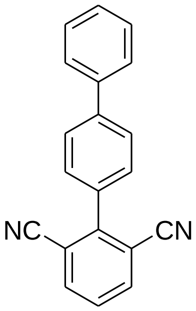
NC
CN

1d

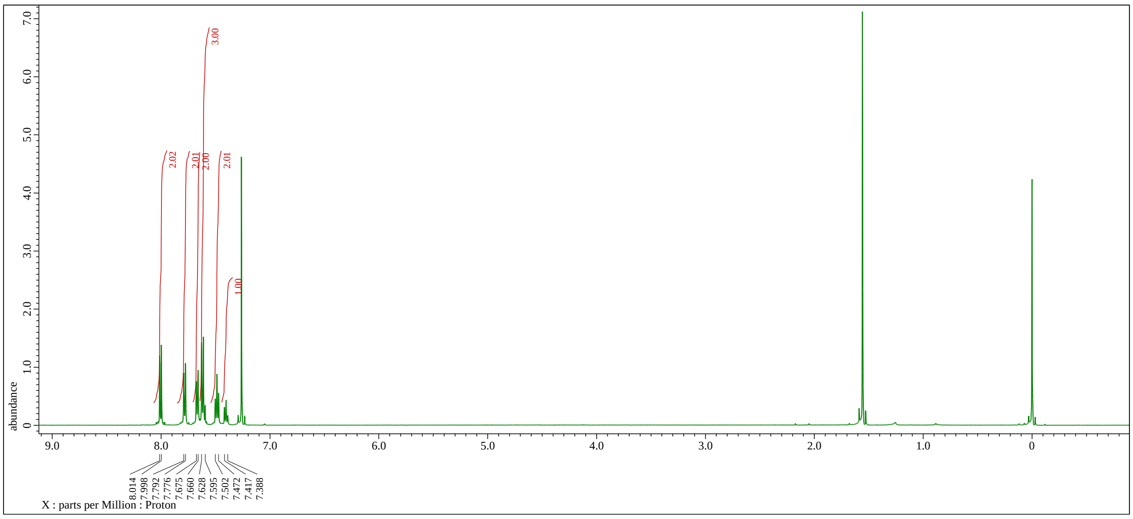
abundance
7.0
6.0
5.0
4.0
3.0
2.0
1.0
0
2.02
2.01
2.00
3.00
2.01
1.00
9.0
8.0
7.0
6.0
5.0
4.0
3.0
2.0
1.0
0
8.014
7.998
7.792
7.776
7.675
7.660
7.628
7.595
7.502
7.472
7.417
7.388
X : parts per Million : Proton

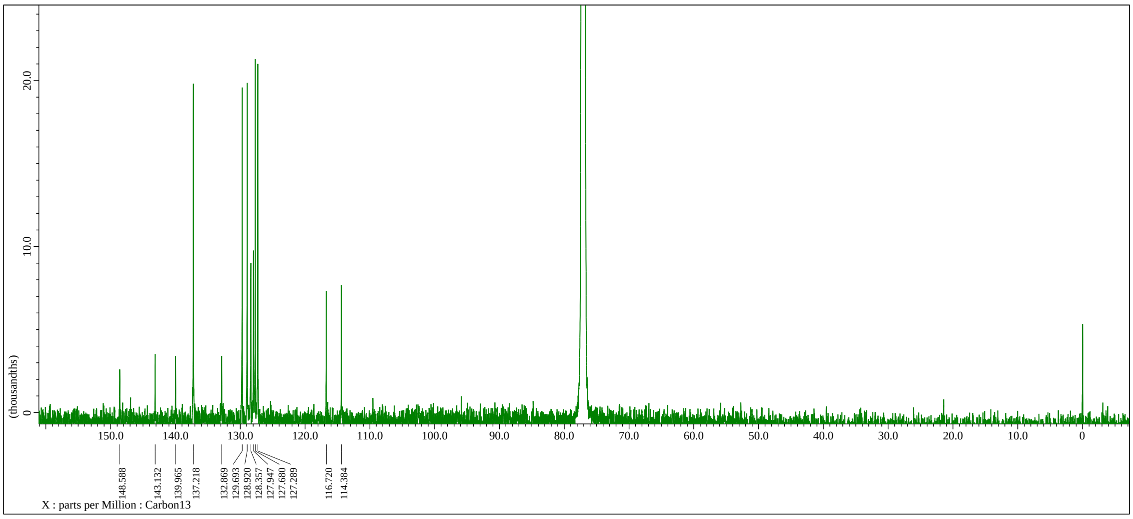
(thousandths)
20.0
10.0
0
150.0
140.0
130.0
120.0
110.0
100.0
90.0
80.0
70.0
60.0
50.0
40.0
30.0
20.0
10.0
0
148.588
143.132
139.965
137.218
132.869
129.693
128.920
128.357
127.947
127.680
127.289
116.720
114.384
X : parts per Million : Carbon13

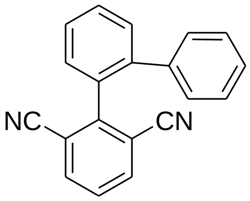
NC
CN

1e

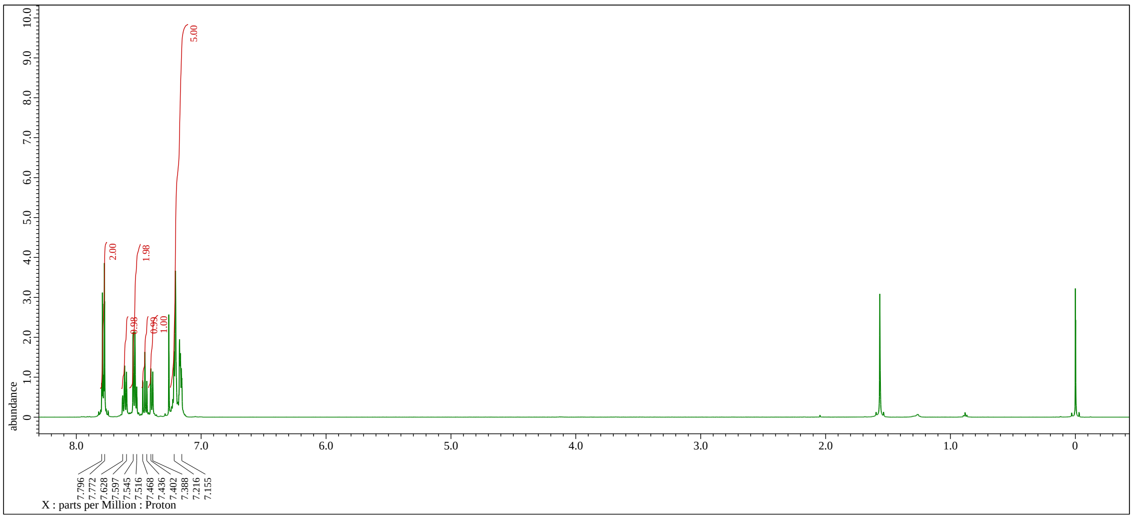
abundance
2.00
1.98
0.98
0.99
1.00
5.00
7.796
7.772
7.628
7.597
7.545
7.516
7.468
7.436
7.402
7.388
7.216
7.155
X : parts per Million : Proton

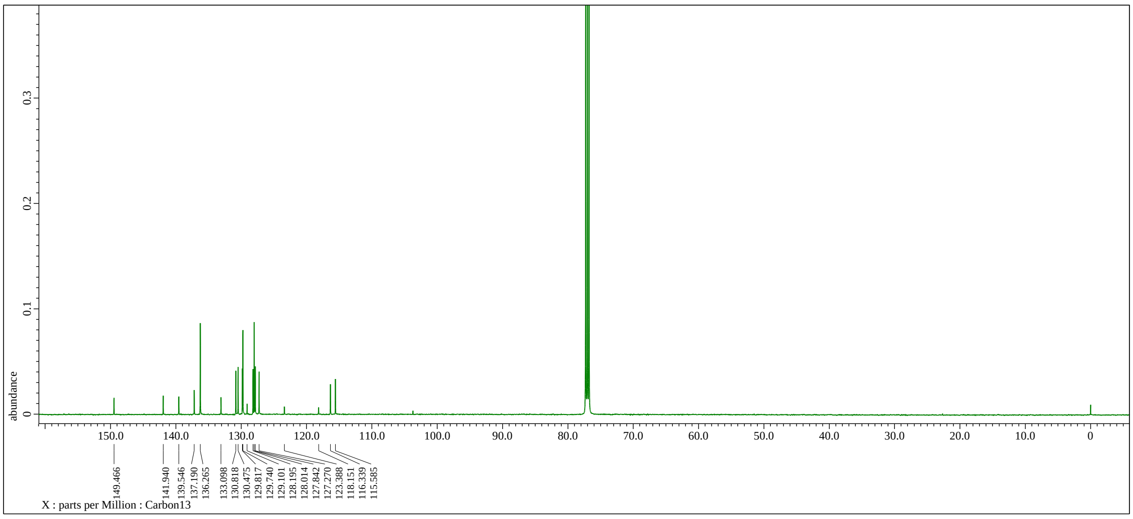
abundance
149.466
141.940
139.546
137.190
136.265
133.098
130.818
130.475
129.817
129.740
129.101
128.195
128.014
127.842
127.270
123.388
118.151
116.339
115.585
X : parts per Million : Carbon13

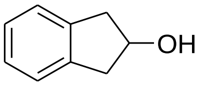

7

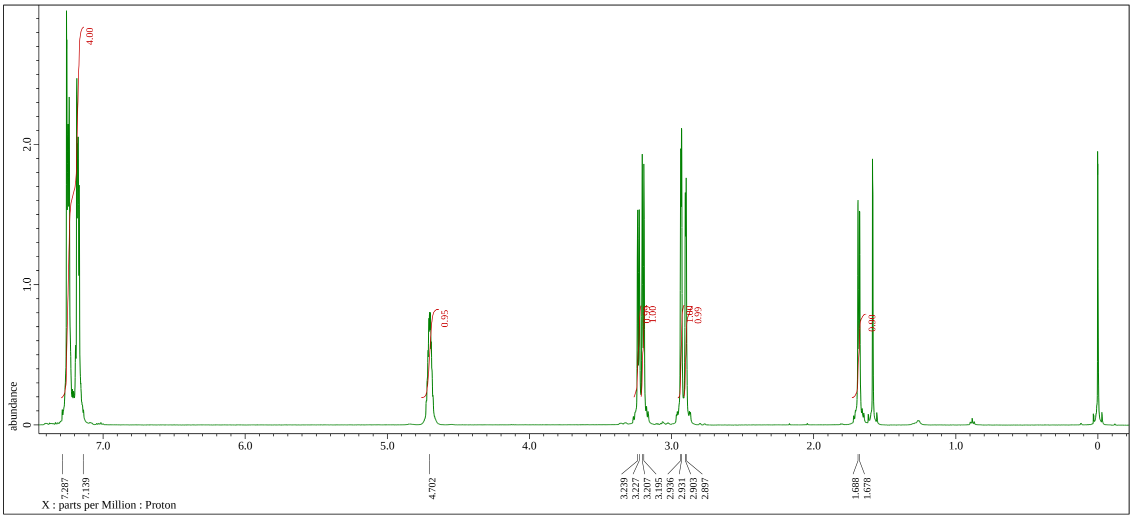

4.00
0.95
0.99
1.00
1.00
0.99
0.90
abundance
0
1.0
2.0
7.0
6.0
5.0
4.0
3.0
2.0
1.0
0
7.287
7.139
4.702
3.239
3.227
3.207
3.195
2.936
2.931
2.903
2.897
1.688
1.678
X : parts per Million : Proton

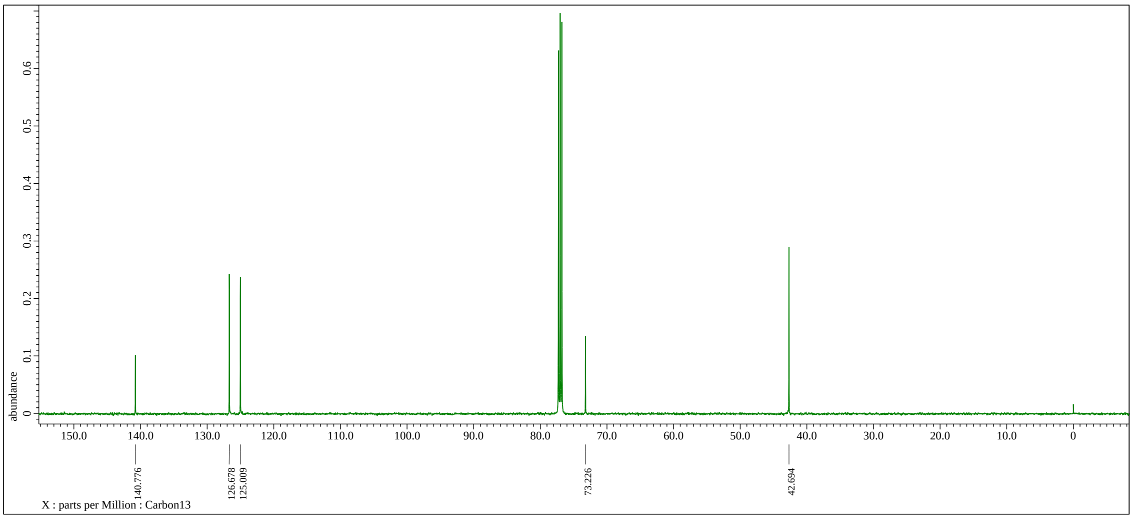

abundance
0
0.1
0.2
0.3
0.4
0.5
0.6
150.0
140.0
130.0
120.0
110.0
100.0
90.0
80.0
70.0
60.0
50.0
40.0
30.0
20.0
10.0
0
140.776
126.678
125.009
73.226
42.694
X : parts per Million : Carbon13

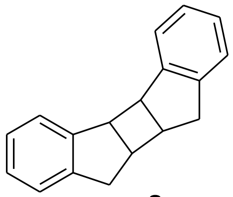

8

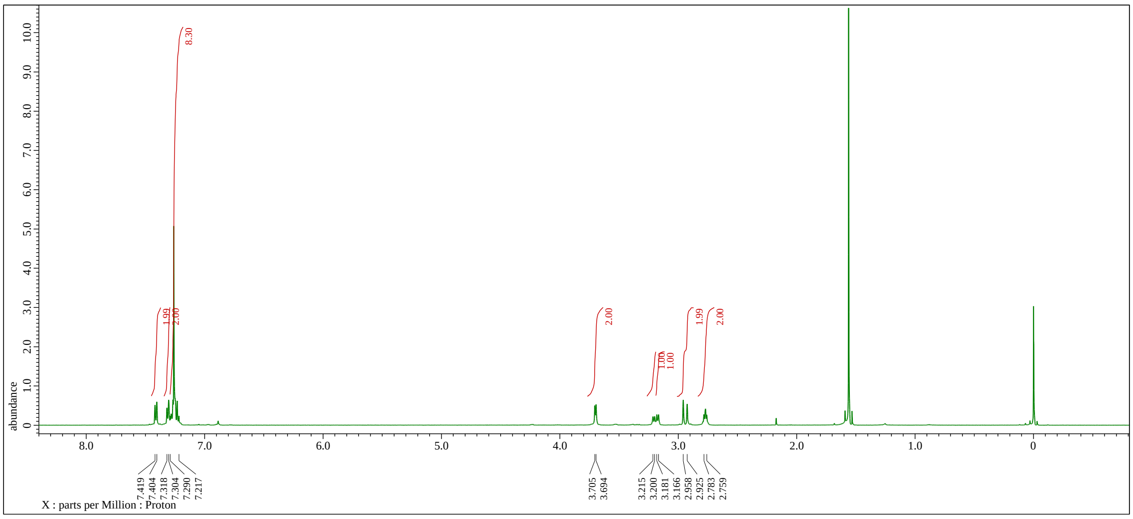

abundance
10.0
9.0
8.0
7.0
6.0
5.0
4.0
3.0
2.0
1.0
0
1.99
2.00
8.30
2.00
1.00
1.00
1.99
2.00
8.0
7.0
6.0
5.0
4.0
3.0
2.0
1.0
0
7.419
7.404
7.318
7.304
7.290
7.217
3.705
3.694
3.215
3.200
3.181
3.166
2.958
2.925
2.783
2.759
X : parts per Million : Proton

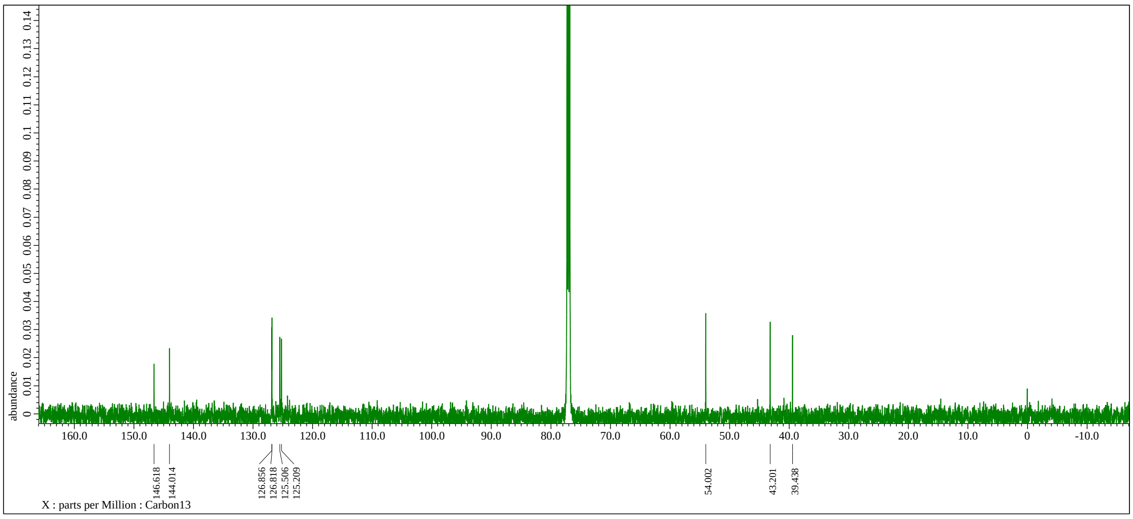

abundance
0.14
0.13
0.12
0.11
0.1
0.09
0.08
0.07
0.06
0.05
0.04
0.03
0.02
0.01
0
160.0
150.0
140.0
130.0
120.0
110.0
100.0
90.0
80.0
70.0
60.0
50.0
40.0
30.0
20.0
10.0
0
-10.0
146.618
144.014
126.856
126.818
125.506
125.209
54.002
43.201
39.438
X : parts per Million : Carbon13

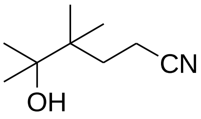
CN
OH

10

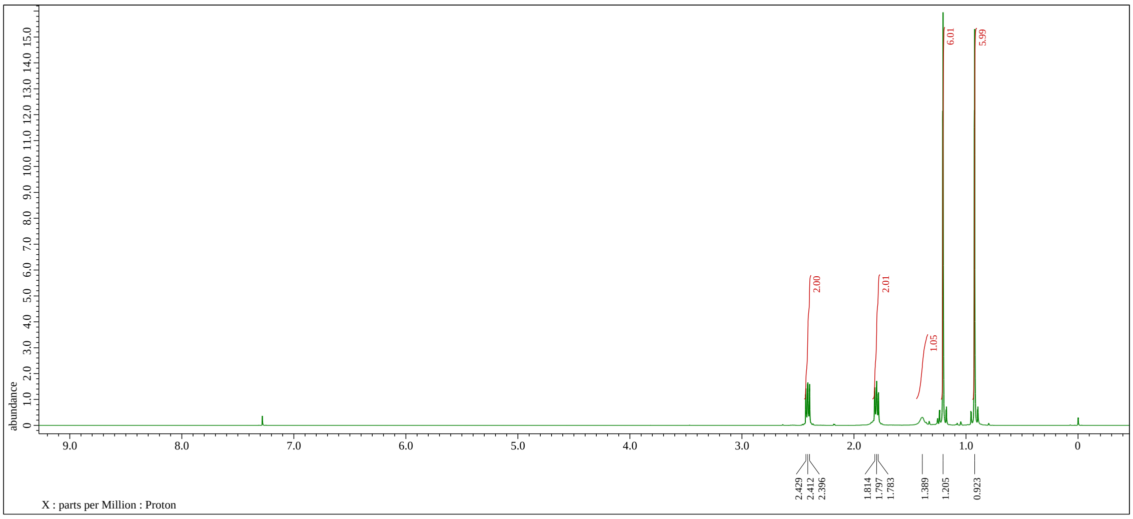
abundance
2.00
2.01
1.05
6.01
5.99
2.429
2.412
2.396
1.814
1.797
1.783
1.389
1.205
0.923
X : parts per Million : Proton

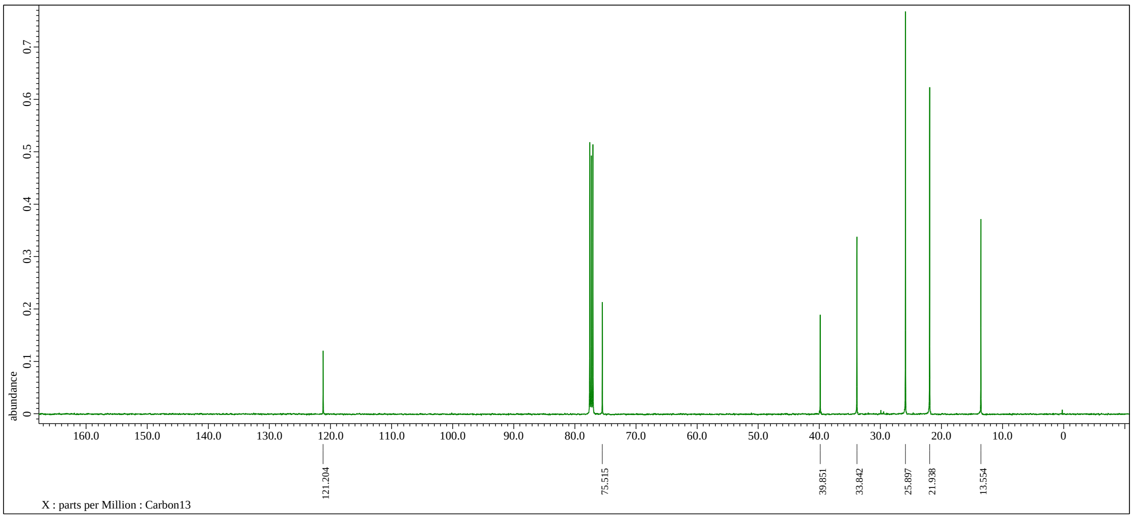
abundance
121.204
75.515
39.851
33.842
25.897
21.938
13.554
X : parts per Million : Carbon13

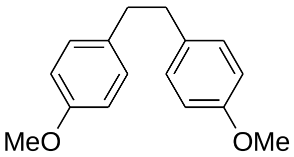
MeO
OMe

11

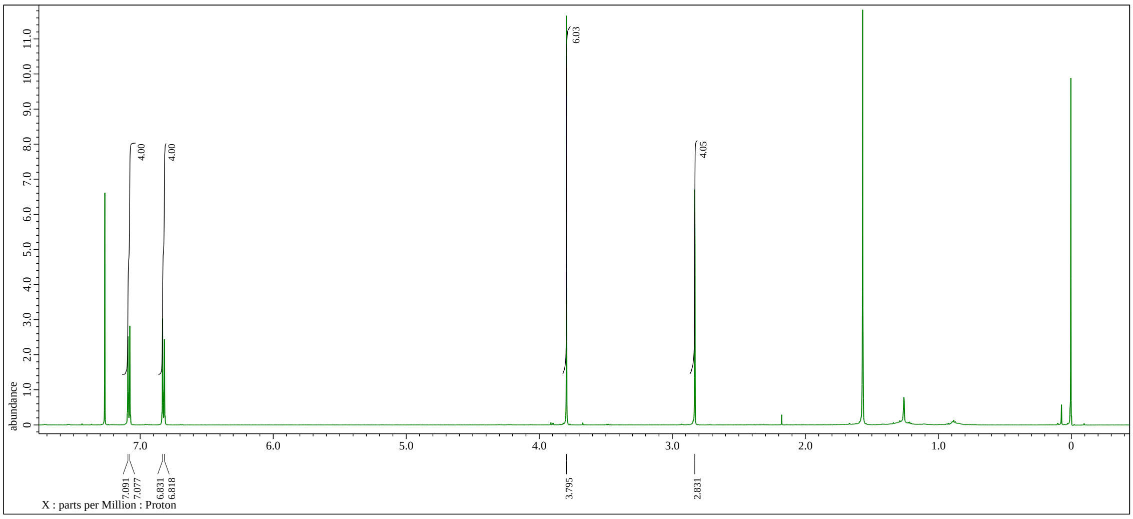
abundance
4.00
4.00
6.03
4.05
7.091
7.077
6.831
6.818
3.795
2.831
X : parts per Million : Proton

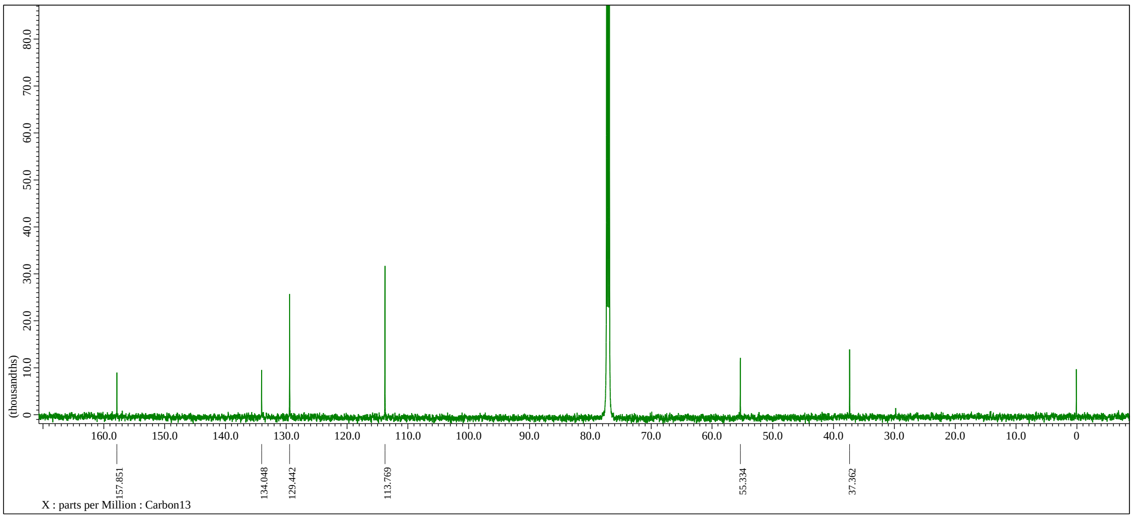
(thousandths)
157.851
134.048
129.442
113.769
55.334
37.362
X : parts per Million : Carbon13
